# Effector Repertoire of *Phytophthora betacei*: In Search of Possible Virulence Factors Responsible for Its Host Specificity

**DOI:** 10.3389/fgene.2020.00579

**Published:** 2020-06-09

**Authors:** Paola Rojas-Estevez, David A. Urbina-Gómez, David A. Ayala-Usma, Natalia Guayazan-Palacios, Maria Fernanda Mideros, Adriana J. Bernal, Martha Cardenas, Silvia Restrepo

**Affiliations:** ^1^Laboratorio de Micología y Fitopatología, Facultad de Ingeniería, Universidad de los Andes, Colombia, Bogota; ^2^Laboratorio de Biología Computacional y Ecología Microbiana, Universidad de los Andes, Colombia, Bogota; ^3^Laboratorio de Interacciones Moleculares de Microorganismos en Agricultura, Universidad de los Andes, Colombia, Bogota

**Keywords:** oomycetes, *Phytophthora betacei*, effector genes, comparative genomics, host specificity

## Abstract

*Phytophthora betacei* is an oomycete plant pathogen closely related to *Phytophthora infestans.* It infects tree tomato (*Solanum betaceum*) in northern South America, but is, under natural conditions, unable to infect potatoes or tomatoes, the main hosts of its sister species *P. infestans*. We characterized, and compared the effector repertoires of *P. betacei* and other *Phytophthora* species. To this end, we used *in silico* approaches to predict and describe the repertoire of secreted proteins in *Phytophthora* species and determine unique and core effectors. *P. betacei* has the largest proteome and secretome of all *Phytophthora* species evaluated. We identified between 450 and 1933 candidate effector genes in *Phytophthora ramorum, Phytophthora sojae, Phytophthora cactorum, Phytophthora parasitica, Phytophthora palmivora, P. infestans*, and *P. betacei* genomes. The *P. betacei* predicted secretome contains 5653 proteins, 1126 of which are apoplastic effectors and 807cytoplasmic effectors. Genes encoding cytoplasmic effectors include 791 genes with an RxLR domain (the largest number known so far in a *Phytophthora* species) and 16 with a Crinkler (CRN) domain. We detected homologs of previously described avirulence gene (Avr) present in *Phytophthora* spp., such as Avr1, Avr3b, Avr4, and Avrblb1, suggesting a high level of effector gene conservation among *Phytophthora* species. Nonetheless, fewer CRN effectors were obtained in *P. betacei* compared to all other *Phytophthora* species analyzed. The comparison between *P. infestans* and *P. betacei* effector profiles shows unique features in *P. betacei* that might be involved in pathogenesis and host preference. Indeed, 402 unique predicted effector genes were detected in *P. betacei*, corresponding to 197 apoplastic effector genes, 203 RxLR cytoplasmic effector genes, and 2 effector genes with CRN domain. This is the first characterization of the effector profile of *P. betacei* and the broadest comparison of predicted effector repertoires in the genus *Phytophthora* following a standardized prediction pipeline. The resultant *P. betacei* putative effector repertoire provides a reasonable set of proteins whose experimental validation could lead to understand the specific virulence factors responsible for the host specificity of this species.

## Introduction

The oomycete genus *Phytophthora* is known for its role as a plant pathogen that infects a wide range of plants of economic importance (Hardham, 2001; Kamoun, 2007). Nowadays there are 142 described species and more than 40 provisionally recognized species as a result of the available genomic technologies and the use of different concepts to define a species within the genus (Grünwald and Flier, 2005; Fry, 2008; Haas et al., 2009; Yang et al., 2017). Notoriously in this group stands *Phytophthora infestans*, a pathogen causing the late blight disease of potato (*Solanum tuberosum*; Fry, 2008). This pathogen is able to infect other members of the Solanaceae family, including tomato (*Solanum lycopersicum*) and tree tomato (*Solanum betaceum*; Mideros et al., 2018). Recently, *P. betacei* was proposed as a new species within the clade 1c using phylogenetic, population genetics and morphological approaches (Mideros et al., 2018). However, *P. betacei* has not been reported infecting potatoes or tomatoes, the main hosts of its sister species *P. infestans* (Mideros et al., 2018). These observations suggest that this species presents host specificity for tree tomato (*S. betaceum*; Mideros et al., 2018). A detailed investigation of the infection cycle of *P. betacei* and *P. infestans* allowed to clearly identify their biotrophic and necrotrophic stages and to notice that *P. betacei* shows a typical hemibiotrophic infection (Guayazán et al., 2017).

Plant pathogens introduce effector proteins inside host plant cells to promote infection (Evangelisti et al., 2017). Effector proteins target different sites in host plant tissues. Some effectors act in the extracellular space, where they interfere with apoplastic plant proteins involved in plant defense; other effectors such as RxLR and Crinkler (CRN) families, translocate inside host cells (Kamoun, 2006, 2007). RxLR effectors are characterized by the presence of a secretion signal peptide followed by a conserved N-terminal domain defined by the RxLR (Arg–Xaa–Leu–Arg) consensus sequence. The RxLR domain is required for the translocation inside plant cells (Whisson et al., 2007) but it is dispensable for the biochemical activity of the effectors when expressed directly inside host cells (Schoebitz et al., 2013). Thus, RxLR effectors are usually short proteins with low similarity in their C terminal. They are localized into diverse subcellular compartments where they interact with plant target proteins playing an important role in the infection process (Vleeshouwers et al., 2008; Oh et al., 2009). Besides that, the LFLAK motif is present in CRN effectors, named after a crinkling and necrosis phenotype caused by some CRN proteins when expressed in plants (Amaro et al., 2017). Critically, expressed mature CRN proteins retained cell death-inducing activity, suggesting functions targeting cytoplasmic host factors, a hypothesis that was confirmed when the translocation activity of CRN, carrying an LFLAK motif at the N-terminus, was demonstrated (Oh et al., 2009; Schornack et al., 2009, 2010; Stam et al., 2013a).

Effector proteins modulate the immune response of the host and enable the infection process (Kamoun, 2007). Thus, in some cases, they may determine the pathogenicity in a susceptible host (Haldar et al., 2006; Ntoukakis et al., 2009). In this sense, *P. infestans* exhibits a high evolutionary potential and its genome shows a discontinuous distribution of gene density. Effector genes, such as members of the RXLR and CRN families, are located in expanded, repeat-rich and gene-sparse regions of the genome, corresponding to highly plastic genomic regions, enriched in transposable elements (TEs; Haas et al., 2009). Raffaele et al. (2010) supported this hypothesis by identifying new previously-overlooked genes involved in virulence of *P. infestans* using a comparative genomics and transcriptomics approach. Then, they provided genomic context to each effector by quantitatively delimiting the gene-dense and gene sparse regions in the *P. infestans* genome. The effector repertoire of *P. betacei* remains unexplored, and there is no information about the factors responsible for its host specificity. Thus, it could be possible that *P. betacei* presents an effector repertoire that allows it to infect only *S. betaceum* and preclude it from infecting the other main hosts of its sister species *P. infestans*.

The objective of this study was to predict and characterize the *P. betacei* repertoire of effector proteins and its genomic context. We compared this repertoire with that of *P. infestans* and other *Phytophthora* species, in order to shed light on potential candidates that might define host range in this clade of the genus *Phytophthora*. This is the first contribution addressed to characterize the effector profile in this new species, therefore, it would be important to describe new possible virulence factors probably responsible for the host specificity of *P. betacei*.

## Materials and Methods

### Proteome and Secretome Predictions

The genome of *P. betacei* (strain P8084; accession number: PRJNA608953) as well as the genomes and annotation information of *Phytophthora ramorum* (strain: Pr102; accession number: GCA_000149735.1), *P. infestans* (strain: Refseq T30-4; Accession number: GCA_000142945.1), *Phytophthora cactorum* (Strain: 10300; accession number: GCA_003287315.1), *Phytophthora sojae* (strain: V3; accession number: GCA_000149755.2), *Phytophthora parasitica* (strain: INRA310; accession number: GCA_000247585.2), and the transcriptome of *Phytophthora palmivora* (strain: LILI; accession number: PRJNA503573) available at the NCBI database were downloaded and used as input to obtain the proteome of each species. Unfortunately, the genome of *Phytophthora andina* could not be included in the analysis due to its genome assembly poor quality. For *P. betacei*, the genome sequence, is the result of a hybrid assembly between Illumina and PacBio data. The annotation information of *P. betacei* was carried out using MAKER2 (Holt and Yandell, 2011) with default settings and using the annotation information of *P. infestans* T30-4 as input (data not published, available upon request).Briefly, the proteomes were predicted using the gffread function with the “coding only” and “only print mRNAs with a full valid CDS” options, this function is available at the cufflinks (v. 2.2.1) package (Trapnell et al., 2012). Next, to obtain the secretome for the six *Phytophthora* species included in this analysis, we based our prediction strategy on the methodology proposed by Evangelisti et al. (2017) and implemented in the SecretSanta pipeline developed by Gogleva et al. (2018) that takes predicted proteomes as input files. This pipeline corresponds to an interface in R (R Core Team, 2018) that uses different tools to allow the prediction of extracellular proteins that are secreted in a classical way. To this end, Gogleva’s pipeline uses (i) SignalP (v. 2.0) (Nielsen and Krogh, 1998), SignalP (v. 3.0) (Bendtsen et al., 2004), and the most recent version SignalP (v. 4.1) (Petersen et al., 2011) to predict signal peptides and cleavage sites with thresholds specific for oomycetes sequences; (ii) TMHMM (v. 2.0) (Krogh et al., 2001) to discard proteins with predicted transmembrane domains; (iii) TargetP (v. 1.1) (Emanuelsson et al., 2000) to select proteins that do not target plastids or mitochondria. Finally, (iv) all proteins with terminal “KDEL” or “HDEL” motifs were removed, because these motifs are known to be ER-retention signals (Evangelisti et al., 2017). It is worth mentioning that we used the transcriptome of *P. palmivora* as a positive control to assure the correct performance of Gogleva’s pipeline. The whole pipeline is depicted in Supplementary Figure S1.

To avoid SignalP output bias, the first resulting set of predicted proteins having a signal peptide was retained. Then, a retrieval of partial proteins was performed, using the function m_slicer which generates sequences with alternative translation start sites based on the assumption that translation start sites might be misclassified in the proteome, which in turn would result in signal peptides also being misclassified. The m_slicer output was used as an input for secretome prediction pipeline (SecretSanta), as described above, we call this set of proteins, rescued proteins (Supplementary Figure S1).

Finally, we removed duplicate sequences using cd-hit (v. 4.6.8) (Li and Godzik, 2006) with a similarity cutoff of 100%. This process resulted in a non-redundant (NR) dataset of putative secreted proteins that was denominated as the secretome.

### Functional Annotation of Secreted Proteins

To annotate the predicted secretome, different approaches were used. Initially we performed a blastp (Altschul et al., 1997) search against the GenBank NR database with an e-value cutoff of ≤10^–6^. In addition, draft functional annotations were assigned for proteins using InterProScan (v. 5.18–57.0) (Jones et al., 2014) with the default parameters. The search was performed using databases of functional domains, as implemented previously (Evangelisti et al., 2017), such as PANTHER, Pfam, Coils, Gene3D, Superfamily, Smart, Pirsf, and Prints (Zdobnov and Apweiler, 2001). Based on the InterProScan output, we performed an analysis of GO (gene ontology) terms annotations. Graphs only show GO terms with a frequency above the 75 quantile of the GO terms count for the three ontology classes (biological process, molecular function, and cellular component). Apoplastic effectors were predicted using ApoplastP (Sperschneider et al., 2018) using default parameters. For the prediction of candidate effectors having RxLR and CRN motifs, the effectR package was used (Tabima and Grünwald, 2019). Once the effector types were determined, we compared the effector profile obtained for *P. betacei* with the effectors reported for the other six *Phytophthora* species.

To validate our secretome predicted proteins, we collected the reported information on the effector profile of the six evaluated species and generated three control databases: (i) database with 1585 effector proteins predicted *in silico*, where 650 proteins are of CRN type (Armitage et al., 2018) and 935 are of RxLR type (Haas et al., 2009). (ii) database with 36 biologically validated proteins, where 16 are CRN-type effectors (Stam et al., 2013b) and 20 correspond to RxLR-type effectors (Wang et al., 2011). (iii) database with *P. betacei* transcripts with (data not published, available upon request). Then, we screened our secretome predicted proteins against the databases (i) and (ii) using blastp (v. 2.6.2) with an e-value threshold of ≤10^–6^. Only hits with an identity percentage greater than 90% and a coverage above 80% were considered.

### Orthology Analysis

Orthologs identification across all the tested *Phytophthora* species proteomes was performed using OrthoMCL (v. 2.0.9) (Li et al., 2003), with default parameters. Next, we selected only those orthologous groups associated with the secretome of each analyzed species. Additionally, upset plots showing the orthologous groups were generated using UpSetPlot library (v. 0.3.0.post3) (Lex et al., 2014). This analysis was also performed to identify orthologous groups of the RxLR and CRN effector proteins from *P. infestans* and *P. betacei*. We obtained a catalog of core effectors and the unique putative effectors present only in the *P. betacei* genome. We defined the core and unique effectors based on the orthologous groups obtained. The unique effectors correspond to singletons (proteins that did not cluster with any other protein from the other species compared). The core effectors correspond to proteins in an orthologous group where proteins from all the species compared are present.

### Genomic Context

Based on the hypothesis regarding the possible importance of gene distribution to identify new effector candidates, we performed a genomic context analysis using the methodology of Raffaele et al. (2010). We designed a custom script in which we implemented the following steps: (i) First, we identified the single-copy core orthologs using the results obtained in the orthology analysis. (ii) Second, to quantitatively evaluate the parameters that allowed segregation between gene dense regions (GDRs) and gene sparse regions (GSRs) in our data, we evaluated different values for the length cutoff (L). L was used to classify the lengths of flanking intergenic regions (FIRs), measured for each gene, as dense, sparse, or in between. For L values between 100 bp and 6 Kbp, we computed the percentage of core ortholog genes falling in GDRs and GSRs of the total genes located in both of these categories. Of the total core ortholog genes, we calculated the percentage of those genes classified as dense. (iii) We computed the segregation rate defined as the difference between the percentage of core ortholog genes in GDRs and the percentage of core ortholog genes in GSRs. (iv) To select the FIRs cutoff L that best classifies the data, we selected the one that maximizes the segregation rate and in which the percentage of core ortholog genes classified as either gene dense or in the boundaries of gene regions (in-between) corresponded to at least 90%.

## Results

### Secretome Prediction

**FIGURE 1:**
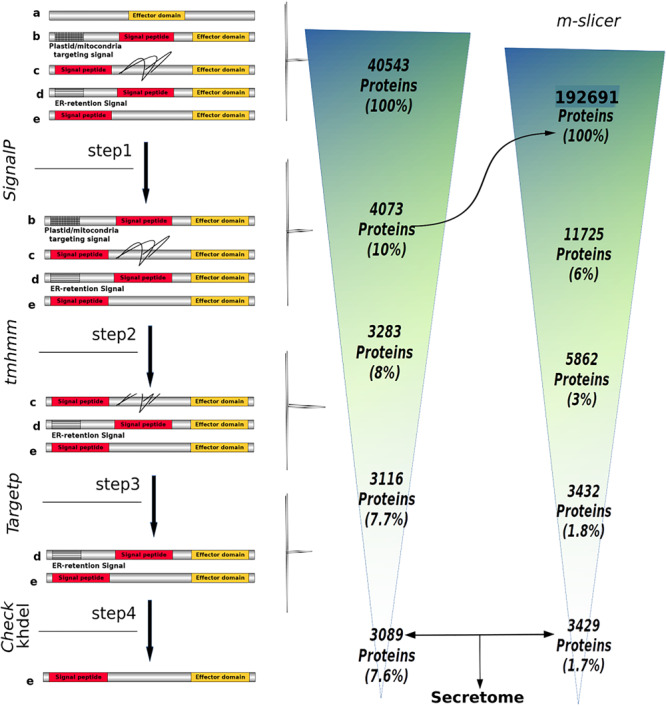
Number of proteins obtained throughout the SecretSanta pipeline used for the prediction of the *Phytophthora betacei* secretome. The secretome prediction pipeline is depicted on the left. The proteins include: (a) proteins without signal peptide, (b) proteins with mitochondria/plastid targeting signals, (c) proteins with transmembrane domains (black lines) located after signal peptide, (d) proteins with short ER lumen retention signals, and (e) proteins with a signal peptide and an effector domain; ensuring the absence of motifs and domains preventing the protein from being secreted or those targeting it to specific organelles. Step 1: identification of short signal peptides at the N-terminal end of a protein using different version of SignalP. In steps 2, 3 and 4, proteins with motifs and domains preventing the protein from being secreted or those that target it to specific organelles are filtered, using TMHMM, TargetP and KHDEL. The number of proteins retained in each step is shown on the right. The m_slicer function that generates sequences with alternative translation start sites, had as input the first resulting set of predicted proteins having a signal peptide (Rightward arrow).

**FIGURE 2:**
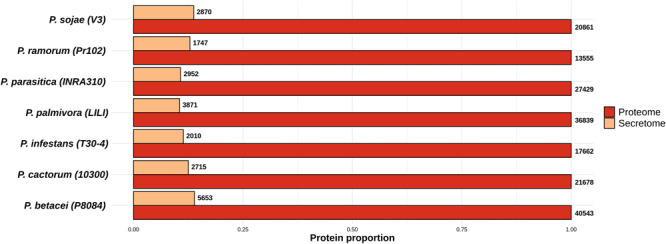
Proportion of the predicted secretomes relative to the proteomes of the *Phytophthora* species evaluated. Proportion of secreted proteins relative to the total proteome for all *Phytophthora* species evaluated. Numbers at the end of each bar indicate the number of proteins predicted in the secretome and proteome of each species. Data were normalized to the size of proteome.

We used an *in silico* secretome prediction pipeline to identify the putative secretome encoded by *P. betacei* (Figure 1). Subsequently, this secretome was compared to those obtained for the other analyzed *Phytophthora* species. The pipeline was implemented to predict signal peptides and cellular localization and to exclude proteins with internal transmembrane domains or an endoplasmic reticulum (ER) retention signal. For the seven species analyzed, secretomes ranged between 10.0 and 13.9% of the total proteome. *Phytophthora betacei* secretome was the largest one with a total of 5653 secreted proteins predicted, corresponding to 13.9% of its proteome (Figure 2 and Supplementary Table S1); *P. palmivora* secretome was represented by a 10.5% of its total proteome; for *P. infestans* and *P. parasitica*, the secretome represented 11.4 and 10.8%, respectively (Figure 2). Protein size distribution analysis of the *P. betace*i secretome revealed that all its proteins (5653) contained between 1999 and 27 amino acids (aa). In the case of *P. infestans* secreted proteins contained between 1562 and 49 aa (Supplementary Table S2). Regarding the seven species, *P. betacei*’s largest predicted protein was above the average of the largest predicted proteins of all the evaluated species. Similarly, this species has the shortest proteins in comparison with the six species evaluated.

### Functional Annotation of the Secretome

**FIGURE 3:**
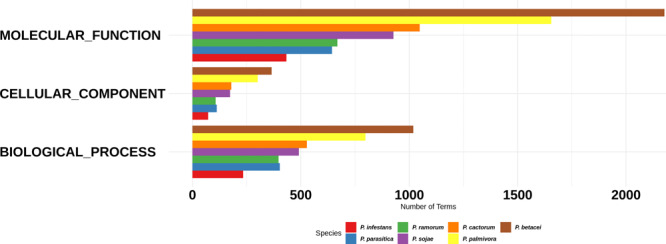
Gene Ontology categories for the *Phytophthora* species secretome. Number of proteins annotated with GO molecular function, GO cellular component, and GO biological process in the secretome. *P. betacei* shows a greater number of proteins associated in each GO category, unlike *P. infestans*, which has a fewer number of associated proteins compared to the other species evaluated.

For the functional annotation of *Phytophthora* secretome, we detected the protein domains using Pfam (v. 32.0) and Superfamily (v. 1.73) HMM model databases and GO terms mapping using InterProScan. A total of 22,226 secreted proteins were obtained for the seven *Phytophthora* species evaluated. Of these secreted proteins we found that 13,704 proteins had at least one predicted domain, and of these, 7234 proteins were associated to at least one GO term (Figure 3). The GO term categorization of the secreted proteins revealed that *P. betacei* had a greater number of proteins in all GO categories than the other analyzed *Phytophthora* species. The “molecular function” category for *P. betacei* presented a greater number of entries when compared to the other six species, particularly to *P. infestans* which showed a lower number of proteins in each GO category with respect to all the evaluated species (Figure 3). As it was the case for the proportion of secreted proteins relative to the whole proteome (Figure 2), the proportions of proteins in each GO categories were similar for *P. palmivora* and *P. betacei* (Figure 3).

**FIGURE 4:**
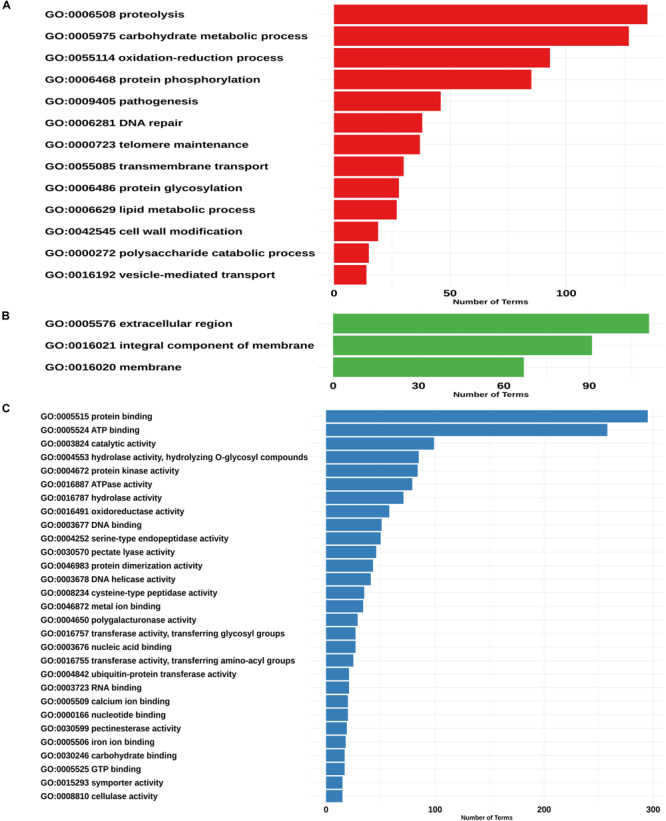
Assignment of GO terms in the *Phytophthora betacei* secretome. Number of proteins annotated in each GO category**. (A)** Proteins annotated with GO biological process **(B)** Proteins annotated with GO cellular component **(C)** Proteins annotated with GO molecular function. We only show those GO terms that are present in the upper 75% of the annotated effector proteins.

We found 52 “biological process” ontologies, 12 “cellular component” ontologies and 84 “molecular function” ontologies shaping the secretome profile of *P. betacei*. Proteolysis (GO:0006508) and carbohydrate metabolic processes (GO:0005975) showed the highest count among biological processes ontologies in the *P. betacei* secretome compared to the rest of the proteomes (Figure 4A and Supplementary Table S3). Other related biological processes are related to Interference with host cell signaling pathways [protein phosphorylation (GO:0006468)] and protein degradation for pathogenesis (GO:0009405). For the cellular component category, organelle and membrane terms were dominant (Figure 4B). The terms that were direct descendants (child terms) of Extracellular region (GO: 0005576) were related to apoplast (GO:0048046), Regarding “molecular function” terms, genes related to binding (GO:0005515; GO:0005524) and catalytic activity (GO:0003824) were highly represented. Remaining terms indicate that *P. betacei* uses plant cell wall degradation activity [cellulase activity (GO:0008810), polygalacturonase activity (GO:0004650), pectate lyase activity (GO:0030570), pectinesterase activity (GO:0030599), and hydrolase activity (GO:0004553)]. Another important molecular function ontology is the endopeptidase inhibitor (GO:0004867), corresponded to Kazal-like serine protease inhibitors (Figure 4C).

### The secretome From *P. betacei* Revealed Both Conserved and Unique Effector Domains

**FIGURE 5:**
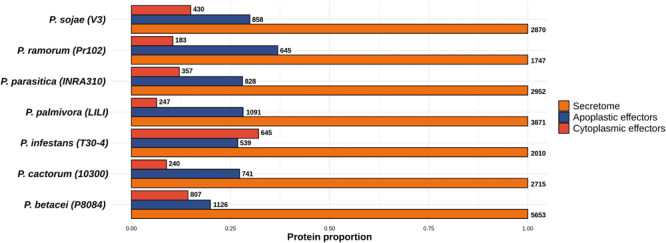
Proportion of predicted effectors relative to the predicted secretome of the *Phytophthora* species evaluated. Proportion of effectors in the secretome. Total amount of proteins predicted as apoplastic effectors and cytoplasmic effectors. Numbers at the end of each bar indicate the number of proteins predicted for the effectors’ categories and for the secretome. Data were normalized to the size of secretome.

In order to identify the effector proteins in the *P. betacei* secretome, we performed an effector domain search for apoplastic and cytoplasmic effectors. Out of the 5653 secreted proteins found in *P. betacei*, 1126 were found to be apoplastic effectors (Figure 5), corresponding to 19.9% of the whole secretome. Comparing with the other *Phytophthora* species evaluated, apoplastic effectors in *P. infestans* represented 26.8% of its secretome, and *P. ramorum* showed the higher proportion with 36.9% of the secretome represented by apoplastic effectors, while *P. cactorum* showed 27.2% of these effectors in its secretome.

**FIGURE 6:**
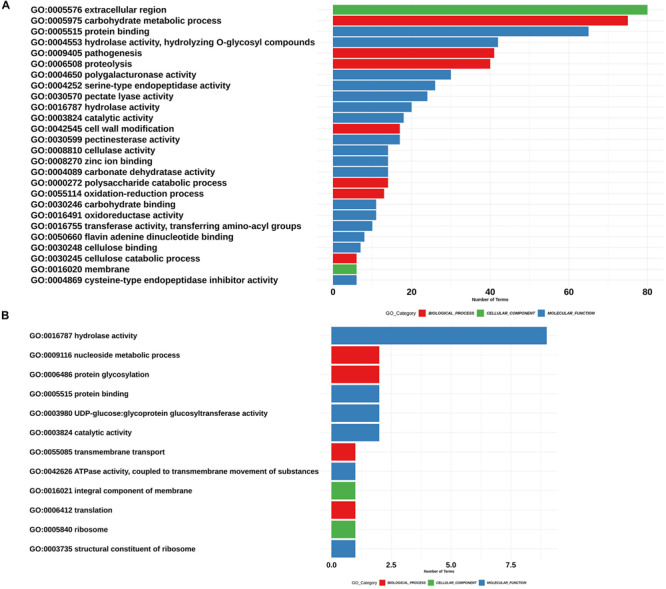
Assignment of GO terms in the *Phytophthora betacei* effectors. Number of proteins associated with each GO Term. The corresponding GO category is shown: Biological process (Red), cellular component (Green) and molecular function (Blue). **(A)** GO terms in putative apoplastic effectors **(B)** GO terms in putative RxLR effectors. We only show those GO terms that are present in the upper 75% of the annotated effector proteins.

For *P. betacei*, 751 (59.6%) of the predicted apoplastic effectors had at least one motif detected by InterProScan search. Of these proteins, 231 (31%) correspond to biological process, 93 (12.%) to cellular component and 427 (54.7%) to molecular function (Supplementary Table S4). Among them, the most representatives are extracellular region (GO:0005576) for cellular component, carbohydrate metabolic process (GO:0005975) for biological process and protein binding (GO:005515) for molecular function (Figure 6A). In addition, we found GO terms associated to infection processes that are highly represented in the set of the predicted apoplastic effectors, such as, pathogenesis and proteolysis. Regarding the blast annotation strategies, we obtained that 538 (47%) out of the 1126 putative apoplastic effectors were annotated when mapped against NR database. Of these, pectin lyase, protease inhibitors, cutinases, Phytotoxic protein (PcF) and necrosis peptide-like proteins (NLPs) domain-carrying proteins previously reported in different *Phytophthora* species were identified. Additionally, we detected elicitins, which can mediate sterol obtention during pathogenesis as well as recognition by some plant cells. On the other hand, we found a high amount of predicted proteins that match with hypothetical proteins related to *Phytophthora* species. It would be interesting to determine the function of these proteins in the future.

**FIGURE 7:**
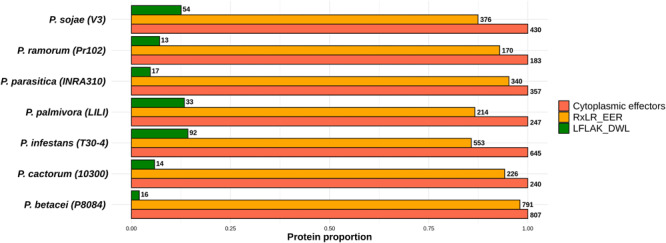
Proportion of effectors with RxLR_EER and LXLFLAK_DWL (CRNs) motifs relative to the total amount of predicted cytoplasmic proteins. Numbers at the end of each bar indicate the number of proteins predicted for each effector category (RxLR and LFLAK). Data were normalized to the number of cytoplasmic effectors.

**FIGURE 8:**
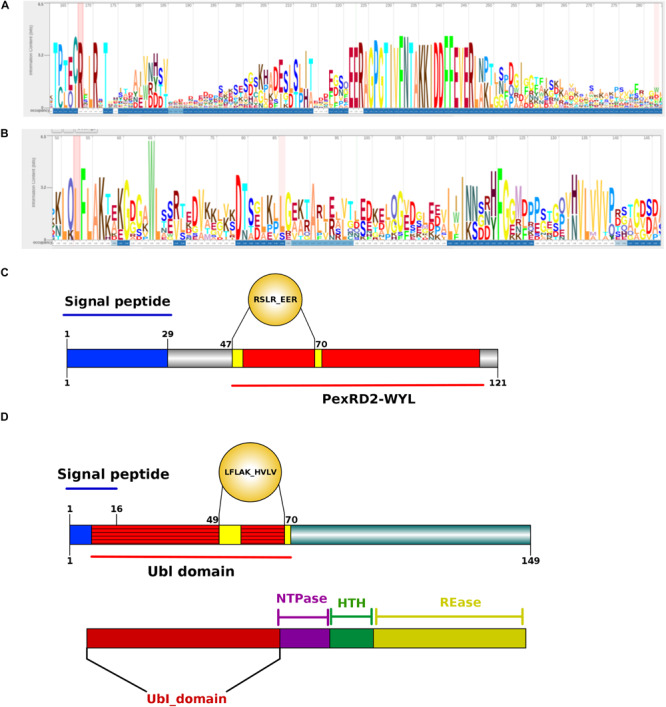
Structure analysis of *Phytophthora betacei* cytoplasmic effectors. **(A)** Architecture of a RxLR_EER putative effector **(B)** Architecture of LFLAK_HVVP CRN putative effector. The bigger the letter, the more conserved the amino acid site. Numbers in the sequence logo are referring to the corresponding positions in the alignment. The C-terminal region is polymorphic. The consensus sequence pattern was calculated using Skylign (V.2.0). **(C)** N-terminal domain consisting of a Signal Peptide (1–29 aa) and a recognized domain in the unique putative proteins with RxLR motif in *P. betacei.* **(D)** N-terminal domain consisting of a Signal Peptide (1–16 aa) and a Ubiquitin like (Ubl) domain recognized in *P. betacei* and *P. infestans* that is thought to be responsible for secretion and translocation into the host cell. At the bottom of the figure, we show the C-terminal domains with distinct domain types as NTPase + HTH + REase.

As mentioned before, cytoplasmic effectors such as RxLR and CRNs are proteins consisting of an N-terminal carrying the conserved domains. For *P. betacei*, we obtained 807 predicted cytoplasmic effectors (12.4% of the secretome) using the effectR package. Of these, 791 correspond to RxLR and 16 to the CRN type (Figure 7). In the alignments of the N-terminal regions of RxLR and CRN effectors, both RxLR_EER and LFLAK_HVL motifs were found (Figures 8A,B, respectively). *P. infestans* showed the highest proportion of cytoplasmic effectors in its secretome, with 32.0%, while *P. ramorum* showed the lowest one with 10.5% (Supplementary Table S1). As expected the majority of N-terminal sequences on the predicted proteins showed a high similarity (Figure 8A) as evidenced in multiple sequence alignment of such effectors. The C-terminal regions, on the other hand, are highly diverse as they are thought to specify effector biochemical functions (Figures 8A,B).

**FIGURE 9:**
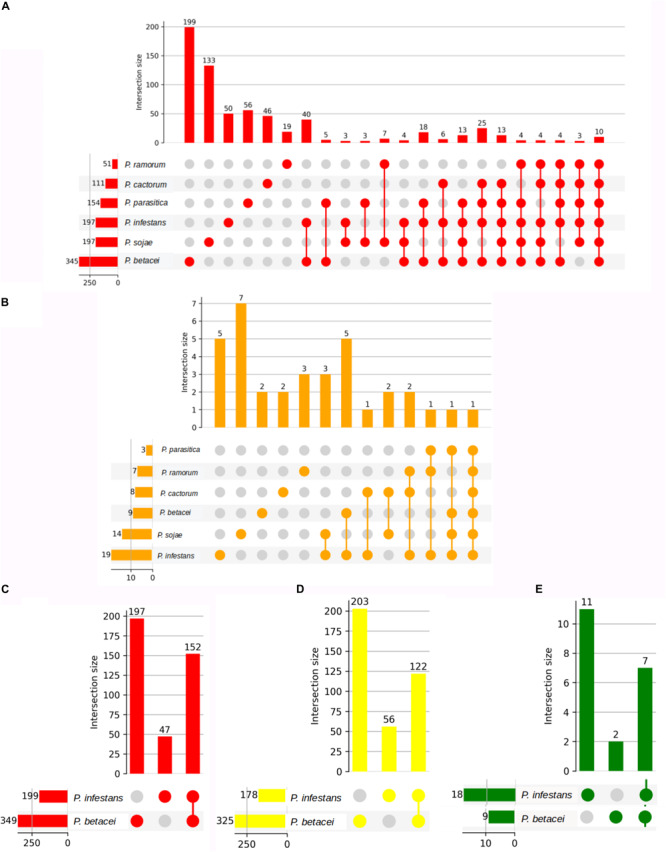
Number of unique ortholog effectors in *Phytophthora* species. Number of effectors from *P. betacei* which are orthologous to those of *P. infestans*. On the bottom left side horizontal bar chart, we show the cluster size of each species. The first columns show singletons effectors present only in each species. Next column shows the number of ortholog effectors shared between the species. **(A)** Orthogroups determined by clustering RxLR effectors in species evaluated without *P. palmivora*. **(B)** Orthogroups determined by clustering CRN effectors in species evaluated without *P. palmivora*. **(C)** Orthogroups determined by clustering apoplastic effectors between *P. betacei* and *P. infestans.* **(D)** Orthogroups determined by clustering RxLR effectors between *P. betacei* and *P. infestans.* **(E)** Orthogroups determined by clustering CRN effectors between *P. betacei* and *P. infestans.*

The putative RxLR effectors were compared among the seven *Phytophthora* species in order to obtain the RxLR core effectors (*P. palmivora* was not included because the predicted effectors were obtained from a transcriptome – see “Materials and Methods”). We obtained 10 RxLR core effectors (Figure 9A) in which the first conserved regions in proteins were found to be situated at the N-terminus, featuring a highly conserved RxLR motif. In addition, we observed that the most frequent amino acids at the x position of the effector motif, were serine, leucine, arginine and lysine (Figure 8A).

With respect to the GO terms annotation of the cytoplasmic effectors, in *P. betacei*, 25 (3.2%) of the predicted RxLR effectors had at least one detected motif in the InterProScan search (Supplementary Table S4). Of these proteins, six effectors (24%) were related to biological processes, two (8%) to cellular components and 17 (68%) to molecular functions. The most representative GO terms included were hydrolase activity (GO: 0016787) for molecular function, nucleoside metabolic process (GO:0009116) and protein glycosylation (GO:0006486) for biological process and two Go terms associated to cellular component, that are, ribosome (GO:0005840) and integral component of membrane (GO: 0016021) (Figure 6B). In addition, the domains Zinc finger FLYWCH-type and PexRD2 WYL were registered as the most frequent ones (Figure 8C). Regarding the blast annotation strategies, we obtained that 322 (40.7%) out of the 791 RxLR effectors were annotated when mapped against NR database. Of these, in the functional characterization of these RxLR core effector, homologs of genes such as Avr1, Avr3b, Avr4, Avrblb1, Avrblb2, and Avrvnt1 were detected, indicating a high level of conservation of RxLR effector genes in *Phytophthora* species (Supplementary Table S4). For both the apoplastic and cytoplasmic effectors predicted for *P. betacei* we found a high amount of hypothetical proteins related to *Phytophthora* species.

On the other hand, we found just one CRN effector shared among all the six species compared (Figure 9B). This CRN core effector featured an Ubiquitin-like (Ubl) domain that is thought to be responsible for secretion and translocation into the host cell. The majority of *Phytophthora* CRN C-terminal regions contained the depicted domain structure (NTPase + HTH + REase) (Figure 8D). Domains as Ubiquitin-like (Ubl_SNRNP25), HAD hydrolase, TIGR01548 family and Elicitin also were found (Supplementary Table S4).

To define core and unique genes between *P. betacei* and *P. infestans*, apoplastic and cytoplasmic effectors in both species were clustered into gene families based on the orthology analysis. In total, 349 apoplastic secreted proteins were predicted for *P. betacei*. Of them, 152 are shared with *P. infestans*, and 197 were considered unique, representing 56% of the predicted apoplastic effectors of *P. betacei* (Figure 9C). Comparison between cytoplasmic effectors of *P. betacei* and *P. infestans*, showed that 62.5% (203) corresponded to *P. betacei* unique RxLR type effectors and 37.5% (122) corresponded to effectors shared between both species (Figure 9D). In the case of CRN type effectors, of nine predicted CRN effectors in *P. betacei*, two were categorized as unique to *P. betacei* representing the 12.5% of the CRN effectors predicted in our species of interest and seven were shared with *P. infestans* (Figure 9E). When performing blastp searches of the effectors categorized as unique of *P. betacei*, most of them show alignments with very low identity percentages and query coverage with hypothetical proteins from other *Phytophthora* species (Supplementary Table S4).

From a total of 205 unique predicted cytoplasmic effector proteins (203 RxLR, 2CRN), 156 mapped on the *P. betacei* transcriptome (data not published, available upon request), of which two of them corresponded to CRN type effectors. A total of 47 out of the 156 predicted effectors expressed in *P. betacei* had at least one annotation based on InterProScan results. The other predicted effectors corresponded to unknown proteins or hypothetical proteins that had not been characterized or linked to known genes.

### Genomic Distribution of *P. betacei* Effectors

**FIGURE 10:**
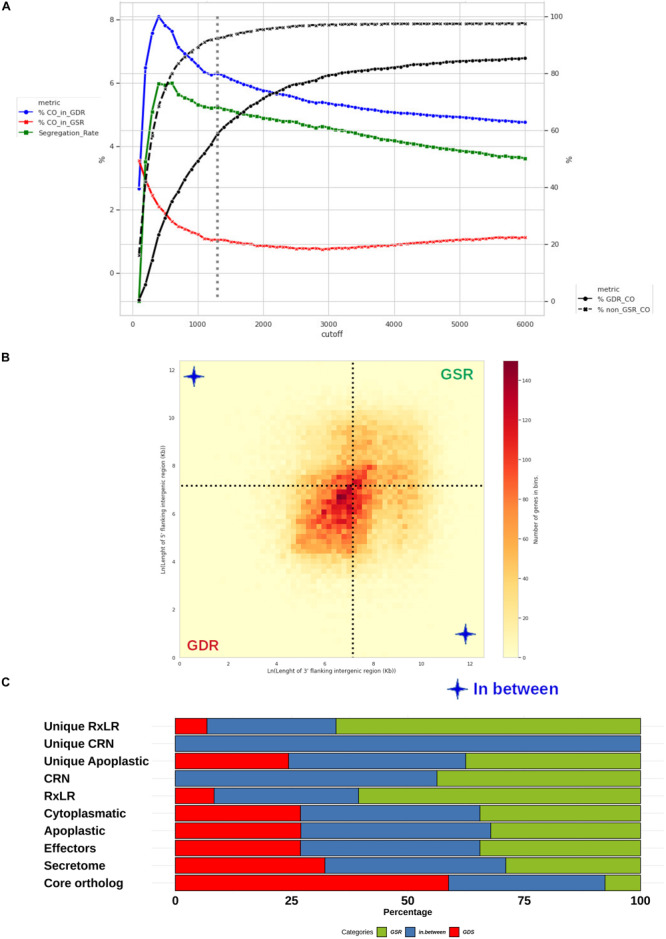
Distribution of *Phytophthora betacei* genes according to the length of their FIRs. Delimitation and effector content of *P. betace*i gene sparse regions (GSRs) **(A)** Simulation of core ortholog gene segregation. Genes with both FIRs longer than a value “L” were considered to be present in a gene-sparse region (GSR), whereas genes with both FIRs below L were considered to belong to a gene-dense region (GDR). The percentage of core orthologs in GDRs (%CO_in_GDR) (blue line), the percentage of core orthologs in GSRs (%CO_in_GSR) (red line) and segregation rate (green line) were calculated for a specific cutoff L value. The core ortholog genes segregation rate was defined as the difference between %CO_in_GDR (blue line) and %CO_in_GSR (red line). The highest core ortholog genes segregation rate in which the percentage of core ortholog genes classified as sparse was less than 10% was obtained for L = 1.3 Kb (dotted lines) **(B)** Distribution of *P. betacei* genes according to the length of their FIRs. All *P. betacei* predicted genes were sorted into 2-variable bins according to their 3’FIR (*Y*-axis) and 5’FIR (*X*-axis). The 1.3 Kbp limit for GSRs genes (dotted lines) delimits three groups of genes: genes found in GDRs, GSRs, and In-between regions. **(C)** Distribution of gene groups into the GDRs and GSRs of *P. betacei*. The proportion of secretome, effectors, RXLR core effector genes, CRN core effector genes and the unique RxLR/CRN that occur in GSRs (green), GDRs (red) and in between (blue) are shown.

We identified 1600 core orthologs for all the sampled species. We established a FIRs cutoff length L = 1.3 Kbp based on the criteria established (see section “Materials and Methods”). At this cut-off, 7.6% of the core orthologs genes were assigned to GSRs, leaving 58.8% of the core orthologs genes assigned to GDRs and a remaining 33.6% of the single-copy core orthologs genes assigned to In-Between regions. At the established cutoff, 6.2% of all the genes in GDRs were core orthologs, 3.1% of all the genes in In-Between regions were core orthologs and, 1.0% of the genes in GSRs were core orthologs (Figure 10A). The GDRs contain 14,924 genes corresponding to 29.8% of *P. betacei* genes. The GSRs contain 11,564 genes representing 26.6% of *P. betacei* genes. The 16,856 genes assigned to the left-superior and right-inferior quadrants correspond to 38.8% of *P. betacei* genes (Figure 10B). The rest of the genes (4.8% of *P. betacei*) were excluded either because they lacked one FIR (located at one border of the scaffold) or they overlapped with other genes. GSRs contain 34.5% of *P. betacei* genes identified in this study as possible effectors. Also 65.5% of the *P. betacei* genes identified as unique RxLR fall in GSRs. This represents an enrichment consistent with previous analysis for other *Phytophthora* species. In particular, genes identified as unique RxLR present a 2.5-fold enrichment compared with the percentage of total genes assigned to GSRs regions. All the genes classified as unique CRN effectors of the species were assigned to In-Between regions, and no CRN effectors were found in GDRs (Figure 10C). We selected 114 apoplastic and 74 RxLR *P. betacei* unique effectors located in GSR regions to be considered as possible virulence factors (Supplementary Table S6).

## Discussion

In this study, we have generated, identified and carefully selected the *P. betacei* secretome to predict the effector profile of this species. We have also identified its main similarities and differences with several *Phytophthora* species and in particular, its sister species *P. infestans*. To accomplish our main objective, we compared different species of the *Phytophthora* genus and generated the catalog of unique and core effectors for each of them. The unique effectors in *P. betacei* will allow us in the near future to experimentally assess if they are involved in host specificity. It is interesting to highlight that, while there are important differences in the number of total proteins among species, the number of secreted proteins showed less differences.

Our results showed that *P. betacei* is the species with the largest proteome and the highest proportion of secretome with respect to its proteome. This species also presents a large number of unique apoplastic and cytoplasmic effectors. These effectors are highly diverse in their sequences and most of them are localized in GSR in the genome. These characteristics suggest that they might be considered as strong candidate virulence factors. Previously, gene distribution (Raffaele et al., 2010; Haas et al., 2013) and gene diversity (Lee et al., 2006; Win et al., 2007; Schornack et al., 2009) were proposed as two main characteristics of effector proteins in *P. infestans*, the sister species of *P. betacei*. In *P. infestans* most known effector genes were found to be in GSRs and presented C-Terminal polymorphic regions, as we found for *P. betacei* in this study (Figures 8A,B).

*Phytophthora* species utilize a diverse range of secreted apoplastic and cytoplasmic effectors to support the infection mechanisms. Effector prediction from genomes or transcriptomes of *Phytophthora* in previous studies have revealed variable numbers of effectors in different species (Jiang et al., 2008; Haas et al., 2009; Evangelisti et al., 2017; Armitage et al., 2018). Our results agree with previous annotations of diverse *Phytophthora* species tested (Supplementary Table S5). We identified a considerably greater number of apoplastic effector candidates (1126) in *P. betacei* than in the other *Phytophthora* species evaluated. Equal or greater numbers of genes encoding elicitins, NLPs, protease inhibitors, cutinases and PcF domain-carrying proteins were identified, the main apoplastic effectors involved in the virulence in *P. infestans, P. sojae*, and *P. cactorum* (Gough et al., 2001; Tian and Kamoun, 2005; Raffaele et al., 2010; Resjö et al., 2017).

Cytoplasmic effectors are defined as those that function specifically within host cells. The past decade of genome sequencing has allowed the identification of hundreds of candidate cytoplasmic effectors in *Phytophthora* genomes. The secretome of *P. betacei* revealed 791 candidate RxLR while for *P. infestans* more than 550 effector genes were predicted (Haas et al., 2009). Proteins in shared ortholog groups between *P. betacei* and *P. infestans* allowed identification of RxLR effector genes fulfilling the typical features of oomycete Avr genes as they encode for modular proteins that contain an N-terminal signal peptide followed by an RxLR motif and a C-terminal effector domain, and occur in gene-sparse regions of the *P. infestans* genome, these effector genes represent key targets for further functional studies.

The CRN protein family is an understudied class of oomycete effectors, difficult to identify and classify. Thus, CRN proteins have hampered functional studies in different oomycetes. Here, we applied a pipeline of CRN prediction, manual verification and mapping studies in catalogs of effectors already reported for the *Phytophthora* species evaluated. This approach improved CRN identification and prediction accuracy for both full length genes and pseudogenes, compared to previously published results (Haas et al., 2009; Zhang et al., 2019). We identified nine CRN effectors in *P. betacei* and when compared to *P. infestan*s, two unique effectors for *P. betacei* were found. Existing descriptions of CRN domain composition and structure, allowed us to identify conserved domains in all *Phytophthora* species analyzed, but also to identify unique ones. One of the two unique *P. betacei* CRN effector (P8084_finalAssembly_32667) has a domain that was previously described in *P. capsici*, as a novel domain called DPA that may have specific roles but that had not been found so far in other oomycetes (Haas et al., 2009; Stam et al., 2013b). This effector could be proposed as a novel type of effector for the clade, and its relevance in pathogenicity needs to be validated. Haas et al. (2009) determined that CRN proteins are modular with domains that execute distinct functions, with a highly conserved N-terminal domain of around 130 amino acids and containing both an LFLAK motif and diversified DWL domains. In our study, the highly conserved HVLVXXP motif marks the end of the N-terminal region as it is considered a recombination hotspot where C-terminal regions, carrying effector functions are linked up (Figure 5A; Haas et al., 2009). In our annotation the unique effectors presented a conserved domain corresponding to a Ubl domain with a beta-grasp Ubl fold, a common structure involved in protein-protein interactions (Amaro et al., 2017).

Effector genes have previously been characterized as showing uneven distributions throughout *Phytophthora* genomes, with measurements of FIRs showing that effector genes are located in gene-sparse regions of the *P. infestans* genome (Haas et al., 2009) and the same scenario was observed for *P. betacei.* This has led to the concept of a “two-speed” genome in these organisms, where different regions of the genome are subject to different evolutionary pressures (Dong et al., 2015). The predicted apoplastic effectors, RxLR and CRN, from *P. betacei* showed increased FIRs distance compared with non-effector genes, supporting that genomic variation in *P. betacei* shows uneven “two-speed” evolutionary rates with the presence of GSRs and GDRs, as previous studies indicated in several *Phytophthora* genomes (Raffaele and Kamoun, 2012; Dong et al., 2015). Unique *P. betacei* effectors that were found to be located in GSRs might be helping the pathogen to colonize and might therefore help explain the higher fitness of this species on tree tomato when compared to its sister species *P. infestans* (Mideros et al., 2018). On the other hand, the unique effectors candidates might also be recognized by potato and tomato and hence could be involved in the lack of ability of *P. betacei* to infect these hosts. Alternatively, the effectorome of *P. betacei* might not be sufficiently effective against crucial targets in tomato and potato cells. Nonetheless functional assays must be performed to validate these hypotheses.

In summary, in the present study we predicted and annotated the putative effector profile of *P. betacei.* We found seven characteristics representative of the *P. betacei* secretome when compared to other *Phytophthora* species: (i) The *P. betacei* proteome has a higher number of proteins. (ii) The proportion of the secretome compared to the whole proteome is higher (13.9%) (iii) Secreted proteins are smaller. (iv) This novel species presents a higher number of RxLR type effectors. (v) The number of cytoplasmic CRN type effectors is lower (vi) One of the CRN proteins shows a domain so far only reported for *P. capsici* (vii) It has 203 cytoplasmic RxLR effectors and 2 unique CRN effectors, and they are polymorphic in their C-terminal region. These characteristics suggest that *P. betacei*, produces a greater number of effectors. Unique effectors that are found in the GSR zones of the *P. betacei* genome are proposed as virulence factors candidates involved in its host specificity, 114 apoplastic and 74 RxLR effectors. These effectors might make it more efficient at colonizing tree tomato when compared to closely related *Phytophthora* species. These effectors might also explain the lack of ability of this species to colonize potato and tomato through recognition by R genes in these plants, which might in turn serve as resistance sources for tree tomato.

## Data Availability Statement

The datasets generated for this study can be found in the repository effectors_predict (https://github.com/ProjasE/effectors_predict).

## Author Contributions

PR-E, DU-G, SR, and AB designed the analyses strategies. PR-E and DU-G performed the analyses. DA-U, NG-P, and MM contributed to the analyses. PR-E, DA-U, AB, MC, and SR wrote the manuscript. All authors contributed to manuscript revision, read and approved the submitted version.

## Conflict of Interest

The authors declare that the research was conducted in the absence of any commercial or financial relationships that could be construed as a potential conflict of interest.

## Supplementary Material

FIGURE S1 Overview of *Phytophthora* species proteome analysis workflow with SecretSanta library developed by Gogleva et al. (2018). The most recent Signalp 4.1 version, was executed by specifying sensitive = TRUE to predict certain classes of secreted oomycete.

TABLE S1 Record of the net values and proportions obtained during the prediction and annotation of the secretome of the different *Phytophthora* species evaluated.

TABLE S2 Size of proteins secreted for each species of *Phytophthora* evaluated.

TABLE S3 Functional characterization of GO found in effector RxLR and CRN in species evaluated.

TABLE S4 Annotation generated for apoplastic, RxLR and CRN effectors using Blastp, Interproscan and GO categorization for core and unique effectors.

TABLE S5 Number of cytoplasmic effects obtained throughout the study and cytoplasmic effectors reported to the *Pythophthora* species evaluated. Here “trCDS” corresponds to the predicted effectors type RxLR and CRN of each species that were predicted using effectR package, and “published” corresponds to the predicted effectors type RxLR and CRN of each species that were predicted and published on NCBI.

TABLE S6 Apoplastic and cytoplasmic effectors candidates as possible virulence factors responsible for *P. betacei* host specificity.
