# Supplementary figures and images for: Effector Repertoire of Phytophthora betacei: In Search of Possible Virulence Factors Responsible for Its Host Specificity
Source: Front Genet. 2020 Jun 9;11:579. doi: 10.3389/fgene.2020.00579 (PMC7295944; doi:10.3389/fgene.2020.00579)

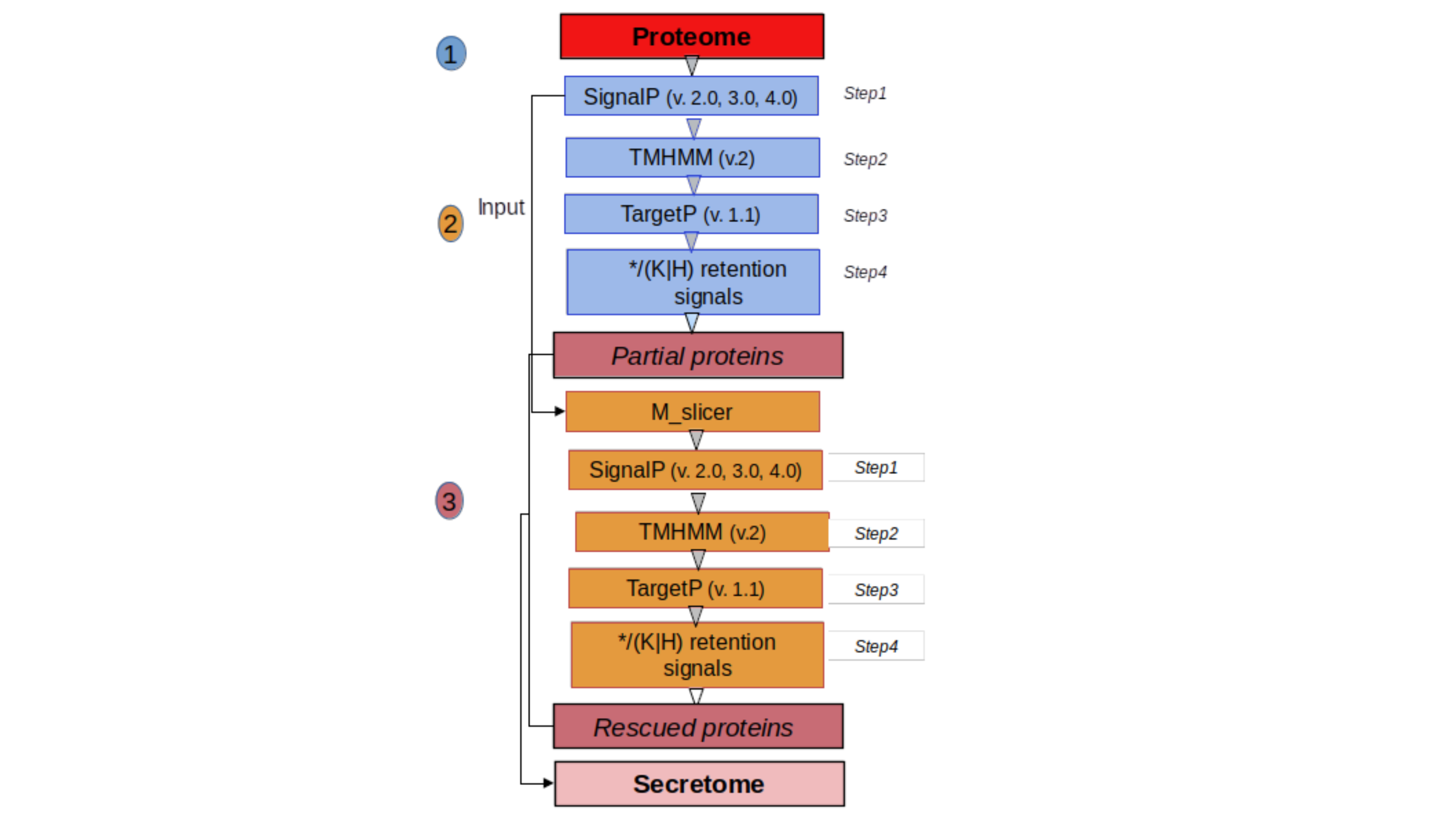

Supplement: FIGURE S1 — Overview of Phytophthora species proteome analysis workflow with SecretSanta library developed by Gogleva et al. (2018). The most recent Signalp 4.1 version, was executed by specifying sensitive = TRUE to predict certain classes of secreted oomycete. [file Image_1.tif]
